# The association between balance and free-living physical activity in an older community-dwelling adult population: a systematic review and meta-analysis

**DOI:** 10.1186/s12889-018-5265-4

**Published:** 2018-04-02

**Authors:** Ilona I. McMullan, Suzanne M. McDonough, Mark A. Tully, Margaret Cupples, Karen Casson, Brendan P. Bunting

**Affiliations:** 1 UKCRC Centre of Excellence for Public Health (NI), Ulster University, Jordanstown, County Antrim, Northern Ireland; 2 Institute of Nursing and Health Research, Ulster University, Jordanstown, County Antrim, Northern Ireland; 3 School of Physiotherapy, University of Otago, Dunedin, New Zealand; 4 UKCRC Centre of Excellence for Public Health (NI); Centre for Public Health, School of Medicine, Dentistry and Biomedical Sciences, Queen’s University Belfast, University Road, Belfast, BT7 1NN Northern Ireland; 5 UKCRC Centre of Excellence for Public Health (NI); Centre for Public Health, School of Medicine, Dentistry and Biomedical Sciences, Queen’s University Belfast, Belfast, Northern Ireland; 6 Institute of Nursing and Health Research, Ulster University, Jordanstown, County Antrim, Northern Ireland; 7 UKCRC Centre of Excellence for Public Health (NI); Psychology Department, Ulster University, Jordanstown, County Antrim, Northern Ireland

## Abstract

**Background:**

Poor balance is associated with an increased risk of falling, disability and death in older populations. To better inform policies and help reduce the human and economic cost of falls, this novel review explores the effects of free-living physical activity on balance in older (50 years and over) healthy community-dwelling adults.

**Methods:**

Search methods: CENTRAL, Bone, Joint and Muscle Trauma Group Specialised register and CDSR in the Cochrane Library, MEDLINE, EMBASE, CINAHL, PsychINFO, and AMED were searched from inception to 7th June 2016.

Selection criteria: Intervention and observational studies investigating the effects of free-living PA on balance in healthy community-dwelling adults (50 years and older).

Data extraction and analysis: Thirty studies were eligible for inclusion. Data extraction and risk of bias assessment were independently carried out by two review authors. Due to the variety of outcome measures used in studies, balance outcomes from observational studies were pooled as standardised mean differences or mean difference where appropriate and 95% confidence intervals, and outcomes from RCTs were synthesised using a best evidence approach.

**Results:**

Limited evidence provided by a small number of RCTs, and evidence from observational studies of moderate methodological quality, suggest that free-living PA of between one and 21 years’ duration improves measures of balance in older healthy community-dwelling adults. Statistical analysis of observational studies found significant effects in favour of more active groups for neuromuscular measures such as gait speed; functionality using Timed Up and Go, Single Leg Stance, and Activities of Balance Confidence Scale; flexibility using the forward reach test; and strength using the isometric knee extension test and ultrasound. A significant effect was also observed for less active groups on a single sensory measure of balance, the knee joint repositioning test.

**Conclusion:**

There is some evidence that free-living PA is effective in improving balance outcomes in older healthy adults, but future research should include higher quality studies that focus on a consensus of balance measures that are clinically relevant and explore the effects of free-living PA on balance over the longer-term.

**Electronic supplementary material:**

The online version of this article (10.1186/s12889-018-5265-4) contains supplementary material, which is available to authorized users.

## Background

Balance, the ability to stay upright and steady whilst moving or stationary, is a complex skill, that requires the contribution from neuromuscular, cognitive, and sensory body systems [1–3]. Good balance is critical for health and well-being in an ageing population. However, whilst many different biological, environmental, socio-economic, and behavioural risk factors have been identified for poor balance [4–10], the ageing process itself is a key risk factor for poor balance. Through disease or degeneration, ageing results in a decline in systems responsible for balance [11], which increases the risk of falling, injury, loss of independence, illness and even mortality in older adults [8, 12–14]. It is estimated that falls affect between 28-35% of those aged 65 years or older, and 32–42% of those aged 70 years or older. Furthermore, the proportion of people aged 60 years or older is growing faster than any other age group and is estimated to reach two billion by 2050, potentially increasing the current human and economic cost of falls by 100% by 2030 [10, 15, 16]. Thus, fall prevention is a key challenge.

A body of evidence derived from clinical trials suggests that exercise, a sub-category of physical activity (PA) that is structured, planned, repetitive, and carried out over a relatively short time frame (from one month to a maximum of 12 months with the most frequent being three months) as outlined by Gillespie et al. (2012) [8] (159 studies; 79,193 participants) and Howe et al. (2011) [13] (94 studies; 9, 821 participants), can maintain balance in higher risk older adults such as those living in institutional care, women, or those with chronic illness (6, 13, 14]. It is also proposed that exercise may even reverse the effects of ageing on balance [17]. Exercise recommendations for older adults at higher risk of falls include individually tailored strength and balance exercise programmes such as Tai Chi programmes [10], and guidelines recommend 120–150 min per week of moderately-intensive PA such as aerobic or muscle strengthening exercise [18–20].

However, whilst evidence suggests that exercise can benefit unhealthy older adults at higher risk of falling, the effectiveness of less intensive PA, that is not defined as exercise, in healthy older adults who are at lower risk of falling is less well understood, and guidelines are less explicit in terms of PA type, duration, and intensity for this lower risk population [10, 20]. Also, statistics suggest that exercise levels in older adults are falling [21, 22], and barriers to exercise for them are identified as: fear regarding personal security; lack of time; lack of social support; lack of interest; lack of appropriate facilities; and environmental issues such as the weather [22–24].

Therefore, this review sought to investigate the effect of free-living PA on balance in an older, healthy adult population (aged 50 years or older), with the aim of informing policy and programmes designed to reduce the fall rate and increase PA levels in older adults. Free-living PA is defined as leisure activity based on personal interests and needs (walking, hiking, gardening, swimming, sport, and dance), travel activity (cycling or walking), occupational activity (labouring, gardening, heavy lifting), or planned exercise in the context of daily, family, and community activities (walking programmes, swimming clubs, Tai Chi clubs) [25–27].

## Methods

### Data sources, searches, and extraction

This review followed the Preferred Reporting Items for Systematic Reviews and Meta-Analyses (PRISMA) recommendations and the Cochrane Handbook for Systematic Reviews of Interventions [28, 29]. To strengthen the methodological approach of the review a protocol was developed a priori using the same guidelines and registered on PROSPERO (CRD42016039114).

Eight electronic databases were searched for relevant articles published up until June 2016 and included (the Central Register of Controlled Trials (CENTRAL), the Cochrane Database of Systematic Reviews (CDSR), the Cochrane Bone, Joint and Muscle Trauma Group Specialised, MEDLINE, EMBASE, CINAHL, PsycINFO, and AMED). Search terms were related to population (healthy, < 50 yrs); intervention (physical activity; activities of daily living, physical mobility, leisure activities, exercise, walking, travel activity, work activity); and outcome of interest (balance, equilibrium, postural control). Details of the MEDLINE search strategy can be found in Additional file 1. In addition, the National Institute for Health Research library [30] and published research on the longitudinal studies of ageing from the English Longitudinal Study of Ageing (ELSA) [31], and the Irish Longitudinal Study on Ageing (TILDA) [32] were screened. Relevant systematic reviews were also manually screened.

Studies were included if they: 1) used an intervention design, or an observational design, 2) included a form of free-living PA, 3) reported a balance outcome measure [33, 34], 4) included a comparison group, 5) included a healthy adult population of 50 years or older, 6) were published in English, 7) were peer-reviewed, and 8) had full text. Excluded were studies including unhealthy older adults with conditions that might impact balance [8]; those studies that met the definition of free-living PA but which took place in a researcher environment or a healthcare facility; and those that included only seated PA [19, 35], interventions such as drug therapy or supplements (e.g. vitamin D), or educational or counselling programmes. Details of excluded studies and reasons can be found in Additional file 2.

Using REFWorks (v. 2.0; ProQuest; Mitchigan, US) [36], titles, abstracts and key words were screened independently by two reviewers against the inclusion criteria. The full-text of eligible articles were then screened independently by two reviewers and data extracted using a pre-tested data extraction form [29]. Discrepancies were resolved by consensus or by third party adjudication. Table 1 shows characteristics of included studies.

**Table 1:** Characteristics of studies exploring the association between Physical Activity and Balance in community dwelling healthy older adults (50 years and over)

Study Author	Study Design	Study Population	Physical Activity measure (type, level)		Outcome measures of balance				Main Finding
		N, Age (mean & range) % female, race, ethnicity, height (m), weight (kg), BMI, education, country, setting, consent	More active (MA) V less active (LA)	Measure, Duration, Intensity	Neuromuscular (Gait (G); Strength (S); Functionality (FU); Flexibility (FL)	Cognitive	Sensory	Other	
Observational Studies:									
Aoyagi et al., 2009 [47]	Prospective cohort 1 year Recruitment: Nakanojo study Conflict of Interest: N/k Source of funding: declared	N: 170 Age: 72.6 ± 4.6 yrs (65-84 yrs) 55% women Height(m): 1.53 ± 0.08 Weight(kg): 54.3 ± 8.6 BMI: 23.3 ± 3.3 Japan Community setting Written informed consent	MA group: 65-74 yr group LA group: 75-84 yr group	Accelerometer MA group: 7190 ± 2491 steps per day LA group: 5482 ± 2829 steps per day	Indirect measure - (G) Walking speed (preferred & maximal) (5 m) (velocity - m/s) - (S) Handgrip test (dynamometer) (force - n) - (S) Isometric knee extension (dynamometer) (torque – N*m/kg) **-** (FL) Functional reach test (distance - m) Direct measure *Static balance test:* Total body stability (eyes open/closed) (sway distance - m)	n/a	n/a	n/a	Measures of physical fitness except handgrip and total body sway were greater for MA group (65-74 yr).
Brooke-Wavell & Cooling, 2008 [50]	Cross sectional 1 time point Recruitment: local bowls clubs; media & friends & family) Conflict of Interest: N/k Source of funding: N/k	N: 74 Age: 68.3 ± 4.65 yrs (60-75 yrs) 100% women Weight(kg): 69.2 ± 10.1 BMI(kg/m^2^): 26.95 ± 3.9 Community setting Written informed consent	MA group: Bowlers LA group: non-bowlers	MA group: 2–3+ hours of PA per week LA group: less than 3 h PA per week	Indirect measure (S) Isometric knee & hip extension (scat & force meter) (force - n) (S) BUA of the calcaneus (Osteometer) (dB/MHz) (FL) TUG (3 m) (time - s) (FL) Range of Motion: shoulder & ankle (goniometer) (degrees°) Direct measure *Static balance test* Total body stability (eyes open/closed (distance - mm)	Reaction time (s)	n/a	Falls	MA group had significantly better postural stability, muscle strength, and flexibility.
Buatois et al., 2007 [51]	Cross sectional 1 time point Recruitment: cohort from a larger study on fall prevention Conflict of Interest: N/k Source of funding: declared	N: 130 Age: 70.3 ± 4.3 yrs 41% women BMI(kg/m^2^): 26.28 ± 3.75 Community setting Written informed consent	MA group: PA -walking, cycling, swimming, gymnastics; PA experience: 28 ± 9.5 yrs LA group: no PA	MA group: 1–2 h per week LA group: no PA	Direct measure Sensory Organisation Test (equilibrium scores and composite score)	n/a	n/a	n/a	Sensory conflicting conditions were more challenging for LA group who swayed more and frequently lost balance than MA group.
Dewhurst et al., 2014 [69]	Cross sectional 1 time point Recruitment: n/k Conflict of Interest: N/k Source of funding: N/k	N: 60 Mean age: 69.36 ± 2.9 yrs (60-80 yrs) 100% women Height(m): 1.58 ± 0.07 Weight(kg): 64.05 ± 8.15 BMI(kg/m^2^): 25.95 ± 3.9 Waist(cm):82.45 ± 9.08 Hip (cm): 102.6 ± 7.62 Waist/hip ratio: 0.80 ± 0.2 Scotland Community setting Written informed consent	MA group: Dancers LA group: Non-dancers	RAPA MA group: 2.5 h hours of PA per week 10 yrs Scottish dance experience LA group: 2.5 h PA per week (no dancing)	Indirect measure (G) Walking speed (preferred/maximum) (6 m) (speed - s) (FL) Timed Up & Go (2.44 m) (time to complete - s) (FL) Range of motion: Chair sit & reach test (distance - cm) (FL) Range of motion: Back scratch test (left/right shoulder) (distance - cm) Direct measure *Static balance test* Total body stability (sway area -cm^2^)	n/a	n/a	n/a	No differences in measures of flexibility between groups. Better results for MA group on measures of TUG, walking and sway.
Fong & Ng, 2006 [52]	Cross sectional 1 time point Recruitment: n/k Conflict of Interest: N/k Source of funding: N/k	N: 48 Age: 55.4 ± 11.5 yrs 50% women Community setting Written informed consent	MA group: tai chi LA group: no tai chi	MA group: 3-6 h per week 1-3 yrs tai chi experience LA group: no tai chi	Indirect measure (FL) Knee repositioning (electrogoniometer) (°degrees; absolute error) Direct measure Tilt board (balance time - s)	Reaction time (electromyography) (ms)	Knee angle repositioning	n/a	MA group had better reaction times, knee joint positioning, and dynamic standing balance measures than LA group.
Fong et al., 2014 [53]	Cross sectional 1 time point Recruitment: martial arts and elderly centres Conflict of Interest: N/k Source of funding: N/k	N: 84 Age: 64.39 ± 11.9 yrs 44% women Weight(Kg): 63.2 ± 11.8 Height(m): 1.60 ± 0.09 BMI(kg/m^2^): 49.3 ± 3.65 Falls: 0.1 ± 0.35 Community setting Written informed consent	MA group: martial arts LA group: no martial arts	MA group: 2 h per week of martial arts Experience: 8 ± 9.9 yrs LA group: no martial arts	Direct measure (S) Bone ultrasound: arm (SOS T & Z scores) Indirect measure (FU) Five times sit to stand (time to complete s) (FU**)** Berg Balance Scale (14 items) (max score 56)	ABC (16 items)	n/a	n/a	MA had better bone strength, lower limb muscular strength and better functional balance than LA group.
Gao et al., 2011 [48]	Cross sectional 1 time point Recruitment: local golf clubs, community centres Authors declare no conflict of interest Source of funding: declared	N: 23 Age: 68.75 ± 6.7 yrs (60-80 yrs) 0% women Height(m): 1.6 ± 0.06 China Community setting Written informed consent	MA group: Golfers LA group: Non-golfers	MLTPAQ MA group: Light =6 Mod. =4 Heavy =1 LA group: Light =10 Mod. = 2 Heavy =0	Indirect measure **(**FL) Functional reach test (forward) (functional reach normalised with body height - %) Direct measure Sensory Organisation Test (somatosensory, visual and vestibular ratios)	MMSE (30 items) ABC (mod.)(16 items)	n/a	n/a	MA group had better balance control, reach, postural control, visual & vestibular inputs. No significant difference between somatosensory ratios between groups.
Gauchard et al., 1999 [54]	Cross sectional 1 time point Recruitment: cohort involved in a study of ageing Conflict of Interest: N/k Source of funding: N/k	N: 40 Age: 72.7 ± 6.5 yrs 70% women Community setting Informed consent	MA group: yoga & soft gymnastics LA group: walking	MA group: 90mins per week LA group: 5 km per week	Indirect measure (S) Knee & ankle extension/flexion, dynamometer (power - Nm/s; strength - Nm) Direct measure *Dynamic balance test* AP stability (eyes open/closed) (foot displacement - FFT; strategy type - Type 1, 2, & 3)	n/a	n/a	n/a	Regular PA improves measures of strength and postural control.
Gauchard et al., 2001 [55]	Cross sectional 1 time point Recruitment: cohort involved in a study of ageing Conflict of Interest: N/k Source of funding: N/k	N: 36 Age: 72.9 ± 6.5 yrs 72% women Community setting Informed consent	MA group: yoga & soft gymnastics LA group: walking	MA group: 90mins per week and 5 km walking per week LA group: 5 km per week	Direct measure *Static balance test* AP (eyes open/closed) (EC/EO ratio) *Dynamic balance test* AP stability (eyes open/closed) (component velocities of nystagmus -left, right, total R-MSCV; L-MSCV; T-MSCV; strategy type Type 1, 2, 3)	n/a	Vestibular tests (caloric/rotational-vestibular reflectivity)	n/a	Inactivity causes poor balance, vestibular hypo excitability and dependency on visual afferent. PA such as yoga improves dynamic postural control.
Gauchard et al., 2003 [56]	Cross sectional 1 time point Recruitment: cohort study of age-related physiology Conflict of Interest: N/k Source of funding: N/k	N: 44 Median age: 73.33 yrs (63-85 yrs) 100% women Community setting Written informed consent	MA group: yoga & soft gymnastics LA group: no PA: walking	MA group: 90 mins per week LA group: n/k	Direct measures *Static balance test* Total body stability (sway distance - m; sway area -cm^2^) AP & ML stability (eyes open/closed) (sway distance - m; sway area - cm°; ratio - EO/EC)	n/a	n/a	n/a	Regular PA increases postural control in older adults. Proprioceptive PA like yoga is more successful in improving static balance.
Gaudagnin et al., 2015 [71]	Cross sectional 1 time point Recruitment: n/k Authors declare no conflict of interest Source of funding declared	N: 24 Age: 67.5 ± 5.5 yrs 100% women Height(m): 1.54 ± 0.06 Weight(Kg): 65.5 ± 10.5 Brazil Community setting Written informed consent	MA group: PA LA group: no regular PA	MA group: at least 150mins per week LA group: no PA	Indirect measure (G) Walking speed (preferred) (8 m) (velocity - m/s)	n/a	n/a	n/a	Active lifestyle improves gait speed.
Gyllensten et al., 2010 [64]	Cross sectional 1 timepoint Recruitment: community centres Authors declare no conflict of interest Source of funding: N/k	N: 44 Age: 69.9 ± 6.85 yrs 82% women Weight(k) 154.8 ± 6.95 Height(m): 1.55 ± 6.95 Hong Kong, China Community setting Written informed consent	MA group: Tai chi LA group: Non-tai chi	MLTPAQ MA group: Light =4 Mod. =17 Heavy =3 LA group: Light =7 Mod. =12 Heavy =1	Indirect measure (FU) Body Awareness Scale- Healthy (BAS-H) (25 items) (FU) Single Leg Jump Test (yes/no; s) Direct measure *Dynamic balance test* Limits of Stability (movement velocity - °/sec; endpoint excursion - %; maximum excursion - %; directional control - %)	MMSE (mod.) (30 items)	n/a	n/a	MA group had better stability limits, increased ability to perform a single leg stance, more stability on landing on one leg, and better body awareness.
Hakim et al., 2004 [70]	Cross sectional 1 time point Recruitment: local tai chi clubs/senior centres Conflict of Interest: N/k Source of funding: N/k	N: 94 Age: 75.2 ± 7.5 yrs (60-96 yrs) 84% women 87% 1 or more chronic conditions 88% independent ambulation Pennsylvania; US Community setting Written informed consent	MA group: Tai chi LA group: No exercise	MA group: 62.5% walk regularly and 100% take a tai chi class 1 or more times per week tai chi experience: mean 5.6 yrs LA group: no tai chi and no walking	Indirect measure (FU) Timed Up & Go (3 m) (time to complete - s) (FU) Chair stand test (30s) (number of full stands) (FL): Multidirectional reach test (distance - inches)	ABC (16 items)	n/a	n/a	MA group have better balance performance, confidence, and multidirectional reach results
Hakim et al., 2010 [57]	Cross sectional 1 time point Recruitment: local tai chi/senior centres Authors declare no conflict of interest Source of funding: N/k	N: 52 Age: 74.46 ± 5.09 yrs 87% women Marital status: Single = 17%; Married = 30%; Divorced = 11% Widowed = 42% 17% comorbidities 37% fall history Community setting Informed consent	MA group: Tai chi LA group: No exercise	MA: 11.66 ± 5.15 (days/month) LA group: 10.73 ± 9.52 (days/month)	Indirect measure (FU) Fullerton Advanced Balance Scale (FAB) (10 items) (FU) Time Floor Transfer test (time to complete - s) (FU) Single leg stance (30s) (balance time - s) (FL) Multidirectional reach test (distance - inches)	ABC (16 items)	n/a	n/a	MA group have better balance performance scores on FAB and multidirectional reach test. No significant differences found on ABC, single leg stance, and Timed floor transfer test between groups
Lu et al., 2013 [65]	Cross sectional 1 timepoint Recruitment: local tai chi clubs/ elderly centres Authors declare no conflict of interest Source of funding declared	N: 58 Age: 73.5 ± 5.15 yrs 72% women Height(m): 1.54 ± 0.80 Weight(kg): 56.95 ± 9.1 Hong Kong, China Community setting Written informed consent	MA group: Tai chi LA group: Non-tai chi	MA group: Light = 4 Mod. =23 Heavy = 1 Minimum of 1.5 h per week tai chi Tai chi experience: 6.7 ± 4.6 yrs LA group: No tai chi: Light = 5 Mod. =25 Heavy = 0	Direct measures *Static balance test* Total body sway (dual and single task) (sway distance - mm; sway area - cm^2^)	MMSE(30 items) Auditory Stroop test (reaction time (s); error rate (%)		n/a	MA group performed better in both stepping down and Stroop tests and so have better postural control and cognitive performance whether there is a single or dual task situation.
Perrin et al., 1999 [72]	Cross sectional 1 time point Recruitment: cohort study of ageing Authors declare no conflict of interest Source of funding: N/k	N: 65 Age: 71.8 s ± 0.8 yrs 66% women France Community setting	MA group: either walking, swimming, cycling, tennis LA group: no PA	MA group: n/k LA group: no PA	Direct measure *Static balance test:* Total body stability (eyes open/closed) (sway velocity - cm/s; sway area - cm^2^) AP/ML stability (eyes open/closed) (sway velocity -cm/s; sway area - cm^2^) *Dynamic balance test:* Tilt board (Short, medium, and long latency responses)	n/a	n/a	n/a	Balance in EO or EC conditions is significantly improved in MA group.
Rahal et al., 2015 [58]	Cross sectional 1 time point Recruitment: geriatrician by anamnesis Conflict of Interest: N/k Source of funding: N/k	N: 76 Age: 73.55 yrs (60-80 yrs) 74% women Community setting Written informed consent	MA group: Tai chi group LA group: Dance group	Measure: n/k MA group: up to 3 h tai chi per week LA group: up to 3 h dance per week	Direct measure *Static balance test:* Modified Clinical Test of Sensory Interaction on Balance (mCTSIB) (sway velocity - °/s) Unilateral stance (sway velocity - °/s) *Dynamic balance test:* Walk across test: (sway speed - cm/s; step width - cm; sway velocity - °/s) Sit to stand test: (sway velocity - - °/s; weight transfer - s)	n/a	n/a	n/a	MA group had reduced postural sway and thus improved static and dynamic balance.
Tsang & Hui-Chan, 2004 [59]	Cross sectional 1 time point Recruitment: tai chi clubs Authors declare no conflict of interest Source of funding declared	N: 47 Age: 69.03 ± 6.37 yrs 0% women Height(m): 1.61 ± 6.45 Weight(kg): 62.65 ± 7.75 Community setting Written informed consent	MA group: Tai chi group Tai chi experience: 8.4 yrs LA group: No exercise group	MLTPAQ MA group: Light =7 Mod. =4 Heavy = 1 PA - Up to 1.5 h p/w LA group: Light = 10 Mod. =2 Heavy =0 Walked/ stretching exercise daily	Direct measure *Dynamic balance test* Limits of stability test (reaction time (s); maximum excursion (%); directional control (%))	MMSE (30 items)	Passive knee joint repositioning test (dynamometer); (absolute angle error - °)	n/a	MA group had better knee joint proprioception and greater limits of stability (dynamic balance).
Tsang & Hui-Chan, 2005 [60]	Cross sectional 1 timepoint Convenience sampling: tai chi clubs and community centres Authors declare no conflict of interest Source of funding declared	N: 48 Age: 70.45 ± 5.55 yrs 50% women Height(m): 1.55 ± 0.07 Weight(kg): 58.1 ± 9.05 Community setting Written informed consent	MA group: Tai chi LA group: No tai chi	MLTPAQ MA group: Light =17 Mod. =5 Heavy = 2 PA Up to 1.5 h per week LA group: Light =21 Mod. =3 Heavy =0 Walked/ stretching exercise daily	Indirect measure (S) Isokinetic knee strength test (dynamometer) (peak torque to body weight ratio) Direct measure *Static balance test* AP & ML body stability (body sway angle °) *Dynamic balance test* AP & ML body stability (body sway angle °)	ABC (16 items)	n/a	n/a	MA group showed better knee muscle strength, less body sway in static standing and perturbed single leg stance and greater balance confidence.
Tsang & Hui-Chan, 2006 [61]	Cross sectional 1 timepoint Recruitment: tai chi clubs/ community centres Conflict of interest: N/k Source of funding: N/k	N: 48 Age: 70.45 ± 5.55 yrs 50% women Height(m): 1.55 ± 0.09 Weight(kg): 58.1 ± 17.5 Community setting Written informed consent	MA group: tai chi group Tai chi experience: mean 8.5 yrs LA group: No tai chi group	MLTPAQ MA group: Light =17 Mod. =5 Heavy =2 PA Up to 1.5 h per week LA group: Light =21 Mod. =3 Heavy =0 Walked/ stretching exercise daily	Direct measure *Static balance test* Total body stability pre-& post vestibular stimulation (eyes open/closed) (sway distance - cm) AP & ML stability pre-& post vestibular stimulation (eyes open/closed) (velocity -cm/s; amplitude°)	n/a	n/a	n/a	MA group have better control of body sway along AP direction.
Tsang & Hui-Chan, 2010 [62]	Cross sectional 1 time point Recruitment: golf clubs/community centres Authors declare no conflict of interest Source of funding declared	N: 23 Age: 68.75 ± 6.7 yrs 0% women Height(m): 1.62 ± 6.95 Weight(kg): 64.05 ± 8.15 Community setting Written informed consent	Ma group: Golfers Golf experience: 15.2 yrs LA group: Non-golfers	MLTPAQ MA group: Light =6 Mod. =4 Heavy =1 PA Up to 1.5 h per week LA group: Light =10 Mod. =2 Heavy =0 Walked/ stretching exercise daily	Indirect measure (FU) Single leg stance (balance time -s) (FL) forward lunge test (average distance of lunge as % of height) Direct measure *Dynamic balance test* AP body stability (body sway angle °)	N/a	n/a	n/a	MA group achieved significantly longer stance duration during single-leg stance, better results on perturbed single leg stance, smaller sway, larger lunge distance onto both legs.
Tsang et al., 2004 [66]	Cross sectional 1 timepoint Recruitment: centres for elderly Conflict of interest: N/k Source of funding: N/k	N: 60 Age: 53.33 ± 3.73 yrs 50% women Height(m): 1.57 ± 0.09 Weight(kg): 58.7 ± 9.7 Hong Kong, China Community setting Informed consent	MA group: Tai chi group Tai chi experience: 7.2 yrs LA group: No tai chi group	MLTPAQ MA group: Light =1 Mod. =15 Heavy =4 PA Up to 3 h per week LA group: Light =0 Mod. =15 Heavy = 5 Walked/or stretching exercise daily	Indirect measure (S) Handgrip test (dynamometer) (strength (Kg)) Direct measure Sensory Organisation Test (somatosensory, visual, vestibular ratios)	MMSE(mod.)(30 items)	n/a	n/a	MA group had better postural control under reduced or conflicting sensory conditions (increased reliance on vestibular and visual systems).
Wayne et al., 2014 [49]	Cross sectional 1 time point Recruitment: N/k Conflict of interest: N/k Source of funding: N/k	N: 87 Age: 63.48 ± 7.63 yrs (50-79 yrs) 66% women White: 86% Non-Hispanic: 98% Education: 18 ± 3.3 yrs BMI(kg/m^2^) 25 ± 3.9 Boston, US Community setting	MA group: Tai chi expert LA group: Tai chi naïve	PASS MA group: 6.0 ± 2.0 (intensity/mins per week) LA group: 4.4 ± 2.2 (intensity/mins per week	Indirect measure (FU) Timed Up & Go (time to complete - s) (FU) Single leg stance (balance time - s) Direct measure *Static balance test* Total body stability (eyes open/close) (sway velocity (mm/s); sway area (mm^2^)) *Dynamic balance test* AP & ML stability (eyes open/closed) (sway velocity (mm/s)	MMSE (30 items)	n/a	n/a	Complexity based measures of sway, single leg stance and TUG are better for MA group.
Wong et al., 2001 [67]	Cross sectional 1 time point Recruitment: tai chi clubs; volunteer group Conflict of interest: N/k Source of funding declared	N: 39 Age: 68.47 ± 5.53 yrs 69% women Weight(kg): 64.73 ± 8.03 Height(m): 1.57 ± 0.08 Taiwan Community setting Informed consent	MA group: tai chi LA group: no tai chi	MA group: tai chi Experience: 15.6 ± 10.5 yrs LA group: no tai chi	Direct measure *Static balance test* Total body stability (eyes open/closed) (max stability - %; sway velocity - °/s) *Dynamic balance test* Total body stability (eyes open/closed) (max stability - %; sway velocity - °/s)	n/a	n/a	n/a	MA group had better postural control than LA group.
Wong et al., 2011 [68]	Cross sectional 1 time point Recruitment: local tai chi clubs Authors declare no conflict of interest Source of funding declared	N: 86 Age: 66.93 ± 5.63 yrs 62% women Weight(Kg): 58.65 ± 8 Height(m): 1.57 ± 0.07 Taiwan Community setting Written informed consent	MA group: tai chi LA group: no PA	MA group: 162mins per week LA group: no PA	Direct measure *Static balance test* Total body stability (eyes open/closed) (max stability - %; sway velocity - °/s; ankle strategy - %) *Dynamic balance test* Total body stability (eyes open/closed) (max stability - %; sway velocity - °/s; ankle strategy - %)	Reaction time (eye/hand) speed - ms)		n/a	MA group showed significantly greater maximal stability, smaller COP velocity, and greater use of ankle strategy, therefore overall better postural control.
Zhang et al., 2011 [63]	Cross sectional 1 timepoint Recruitment: local tai chi/ walking groups Authors declare no conflict of interest Source of funding declared	N: 30 Age: 65.7 ± 4.9 yrs 100% women Community setting Written informed consent	MA group: Tai chi group LA group: Walking group	MA group: 7 h per week of tai chi 8.2 yrs tai chi experience LA group: 7 h per week of walking 8.8 yrs walking experience	Indirect measure - (FU) Single leg stance (time spent on one leg during walking (s)) - (G) Walking speed (preferred) (velocity (m/s)	n/a	n/a	n/a	MA group have better movement control but LA group have better results on single leg stance measures.
**RCT studies:**									
Paillard et al., 2004 [73]	RCT Baseline & post 12 weeks Randomised but not specified Conflict of interest: N/k Source of funding: N/k	N: 21 Age: 66.15 ± 2 yrs (63-72 yrs) 0% women Weight(kg): 74.8 ± 6.7 Height(m): 1.71 ± 0.05 Community setting Written informed consent	Intervention group: 3 months walking programme Control: no walking programme	Baseline measure: n/k MA group: up to 5 h of walking per week for 3 months LA group: up to 3 h per week no walking programme	Indirect measure (G) Walking speed (preferred) (velocity - m/min) Direct measure *Static balance test* Total body stability (eyes open/closed) (sway distance -- mm; sway area -mm^2^; speed variation; ratio - EO/EC*100) AP & ML stability (eyes open/closed) (distance - mm; sway area - mm^2^) *Dynamic balance test* ML stability (eyes open/closed) (position°; amplitude°; spectral energy- %)	n/a	n/a	n/a	12 week walking programme can improve postural control whilst moving but not when static.
Santos Mendeset al., 2011 [74]	RCT Baseline & post 4 months stratified by sex & randomised Conflict of interest: N/k Source of funding: N/k	N: 30 Age 68.7 ± 3.5 yrs 60% women Weight(kg): 66.9 Height(m): 1.69 Community setting	Intervention group: 4 months walking programme Control: no PA	MA group: 1 h per week for 4 months LA group: no PA	Direct measure *Static balance test* Total body stability (8 positions) (Static Balance Index) *Dynamic balance test* Total body stability (2 tests - hurdle obstacle; sit down and stand up from chair) (Dynamic Balance Index)	n/a	n/a	n/a	Walking is beneficial to both dynamic and static balance.
Wayne et al., 2014 [49]	RCT 3 time points: Baseline, 3 months, 6 months Recruitment: N/k Conflict of interest: N/k Source of funding: N/k	N: 60 Age: 64.19 ± 7.72 yrs (50-79 yrs) 67% women White: 92% Non-Hispanic: 98% Education: 17 ± 3 yrs BMI(kg/m^2^): 26.5 ± 5.5 Boston, US Community setting	MA group; Tai chi expert 6 months tai chi LA group: Tai chi naïve	PASS MA group: 4.0 ± 2.0 (intensity/mins per week) LA group: 4.0 ± 2.0 (intensity/mins per week	Indirect measure (FU) Timed Up & Go (time to complete -s) (FU) Single leg stance (balance time - s) Direct measure *Static balance test* Total body stability (eyes open/close) (sway velocity - mm/s; sway area - mm^2^) Dynamic balance test AP & ML stability (eyes open/closed) (sway velocity - mm/s)	MMSE (30 items)	n/a	n/a	MA group had no significant short term effects from being more active based on traditional COP measures, but some increases in body sway in complexity COP measures (AP and ML eyes closed) correlated to practice hours.
Yang et al., 2007 [75]	RCT Baseline, 2 month, 6 month Randomisation program for 4 locations Conflict of interest: N/k Source of funding: N/k	N: 49 Age: 80.55 ± 8.49 yrs (60-97 yrs) 80% women Retirement home (76%)	MA group: 2 months Tai chi LA group: no tai chi	Measure: n/k MA group: 3 h tai chi per week for 2 months LA group: usual activity 3.67 ± 2.38 h per week	Indirect measure (FU) Berg Balance (baseline only) Direct measure Sensory Organisation Test (somatosensory, visual & vestibular ratios) Base of support (area - cm^2^; feet opening angle °)	n/a	n/a	n/a	MA group have better SOT vestibular results and greater Base of Support measures but no differences for SOT visual ratios or feet opening angle between groups.

Risk of bias assessment was carried out independently by two reviewers, trialled with a small number of studies to check for understanding, and disagreements were resolved by consensus or third-party adjudication. The Cochrane Collaboration tool was used to assess the quality of included intervention studies [29] by considering their internal validity and risk of bias. The approach considers studies are low risk of bias where risk is low across all domains or most information was from studies at low risk; unclear risk where risk is unclear across all domains or most information was from studies at unclear risk; and high risk of bias where one or more domains were high risk or the proportion of information from studies at high risk was sufficient to affect the interpretation of the results. Observational studies were assessed using a variation of the Newcastle Ottawa Scale (NOS) [37–40], and in the absence of formal threshold scores for rating quality [40] studies were rated as high risk of bias if scored four stars or below, and low risk of bias if scored five stars and above (maximum stars possible was ten).

### Data synthesis and analysis

Data were grouped by study design [41], by PA type [42] and then according to balance outcome measure (direct or indirect) [13, 33]. Where data were available and appropriate as per the guidelines outlined by the *Cochrane Handbook for Systematic Reviews of Interventions* [29] a statistical analysis was conducted in RevMan [43] where standardised mean values (95% confidence intervals (CI)) for balance outcomes between more active and less active groups were compared. Where studies involved multiple intervention groups and more than one group met the inclusion criteria, PA interventions were only compared to minimal intervention controls to avoid double counting [44], in accordance with Ainsworth et al.’s Compendium of Physical Activities’ [45]. Additionally, where studies included groups that compared PA levels by gender or age rather than by ‘less’ or ‘more’ PA, then where possible, these groups were combined [29]. Due to the statistical and clinical heterogeneity in the balance measures being combined a random-effects model was used to pool the analyses, and heterogeneity was considered large where *p* < 0.1, and the I^2^ > 50% [29]. Funnel plots that included effect size and standard error were used to examine asymmetry and to assess reporting bias. Post-hoc sensitivity analyses were carried out to assess the possible influence of risk of bias and heterogeneity on the robustness and overall validity of the results where studies were excluded that met high risk of bias criteria (e.g. observational studies with 4 stars or below on NOS; RCTs identified as high risk according to Cochrane’s risk of bias tool).

Where insufficient data were available to complete a meta-analysis the data were synthesised qualitatively using a best evidence synthesis advocated by van Tulder et al. [46] where evidence is considered 1) strong; consistent findings in multiple RCTs assessed as having low risk of bias; 2) moderate; consistent findings in one RCT assessed as having low risk of bias, and one or more RCTs assessed as having high risk of bias, or by generally consistent findings in multiple RCTs assessed as having high risk of bias; 3) limited or conflicting evidence; only one RCT (assessed as having either a low or high risk of bias), or inconsistent findings in multiple RCTs; and 4) no available evidence; no published RCTs that have assessed interventional effect.

## Results

**Fig. 1:**
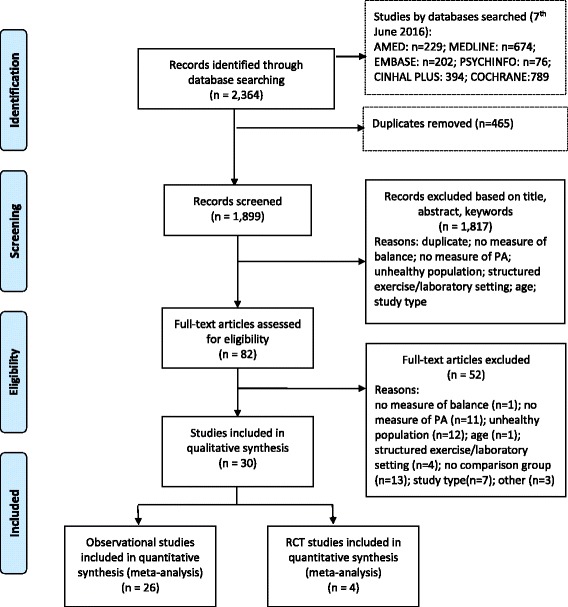
Prisma flowchart

A total of 2364 articles were identified by the search strategy. From the title, abstract, and keywords, two reviewers independently identified 82 relevant studies for full text review. From the full text review, 52 were excluded resulting in 30 papers being reviewed (*n* = 1547 participants). The process, including reasons for exclusions, is shown in Fig. 1 [28].

### Observational studies

#### Design, sample size, and location

Twenty-six studies were observational (one prospective cohort [47], and 25 cross sectional). Sample size ranged from 23 [48] to 170 [47] with an average of 54 participants, but only one study carried out a sample size calculation [49].

Fourteen studies did not specify study location [50–63]; one study was carried out in Japan [47]; four in China [48, 64–66]; two in Taiwan [67, 68]; one in the UK [69]; two in US [49, 70]; one in Brazil [71]; and one in France [72].

#### Participants

Participants across all studies were defined as healthy and resided in the community (62% women; mean age = 66.93 years). Age groups included were: 50–60 years in two studies [52, 66]; 61–70 years in 15 studies [48–51, 53, 59–64, 67–69, 71] and 71 years or over in eight studies [47, 54–56, 58, 65, 70, 72].

There was a lack of demographics in included studies where only one study reported marital status [57], and one study reported ethnicity and education [49].

#### Physical activity

All PA interventions were land based except for two studies that included mixed PA with a component of swimming [51, 72]. Sixteen studies included 3D PA (e.g. dance and tai chi) [42] (*n* = 842 participants), and ten included ‘General’ PA (e.g. walking, cycling) [42] (*n* = 505 participants). Only one study used an objective measure of PA, an accelerometer, measuring steps per day [47], whilst nine used a variety of validated questionnaire based measures (e.g. Rapid Assessment of Physical Activity (RAPA), Physical Activity Status Score (PASS), Minnesota Leisure Time Physical Activity Questionnaire (MLTPAQ) [48, 49, 59–62, 64, 66, 69], and 16 did not specify the tool used [50–58, 63, 65, 67, 68, 70–72].

All studies included a less active group and a more active group and long-term practice of PA ranging from one to 21 years and over, with two identifying one to five years [47, 52]; eight identifying six to ten years [53, 59, 61, 63, 65, 66, 69, 70]; one identifying 11–15 years [62]; one identifying 16–20 years [67]; and one identifying 21 years and over [51]. Thirteen studies did not specify PA duration [48–50, 54–58, 60, 64, 68, 71, 72].

#### Balance

Overall, studies included multiple balance measures, except for three that included only one measure [51, 59, 71]. Sixteen studies included indirect measures relating to the neuromuscular system (*n* = 961 participants) [47–50, 52–54, 57, 60, 62–64, 66, 69–71]. Thirteen studies included indirect measures of cognitive function (*n* = 805 participants) [48–50, 52, 53, 57, 59, 60, 64–66, 68, 70]. Only three studies included any sensory system measures (*n* = 131 participants) [52, 55, 59] and these included proprioception measures. Only one study [50] reported fall rate. Some studies met our inclusion criteria but were excluded from the analyses due to inadequate data and the authors provided no further information on request (*n* = 159 participants) [56, 58, 67]. Results were estimated from graphical information in seven studies (*n* = 429 participants) [51, 52, 54, 55, 68, 71, 72].

##### Secondary outcome measures

Three studies used the Sensory Organisational Test (SOT) [48, 51, 66] (*n* = 139 participants). Force platforms for the measurement of sway for static or dynamic balance were used in 17 studies (*n* = 1028 participants) [47–50, 55, 56, 58–62, 64, 65, 67–69, 72]. The ability to maintain balance whilst standing on a tilt board was measured in one study (*n* = 48 participants) [52].

#### Quality

Table 2 presents a summary table of the risk of bias of included observational studies and shows that in general studies were of moderate quality (*n* = 14 studies). All studies rated poor in terms of comparability of participants; the majority (*n* = 14 studies) failed to provide details relating to selection process, but the measures of balance included in studies were validated and stated in the main objective.

**Table 2:** Newcastle-Ottawa Scale risk of bias assessment of observational studies

Study	Selection (max. 5 stars)	Comparability (max. 2 stars)	Outcome (max. 3 stars)	Total (max. 10 stars)
Aoyagi et al., 2009 [47]	***	*	***	7
Brooke-Wavell & Cooling, 2008 [50]	*	*	***	5
Buatois et al., 2007 [51]	*	*	***	5
Dewhurst et al., 2014 [69]	**		**	4
Fong & Ng, 2006 [52]	*	*	***	5
Fong et al.,2014 [53]	*	*	***	5
Gao et al., 2011[48]	***	*	***	7
Gauchard et al., 1999 [54]	*		***	4
Gauchard et al., 2001[55]	*		***	4
Gauchard et al., 2003[56]	*		**	3
Gaudagnin et al., 2015	*		***	4
Gyllensten et al., 2010 [64]	***	*	***	7
Hakim et al., 2004[70]	*		***	4
Hakim et al., 2010 [57]	*		***	4
Lu et al., 2013[65]	*	*	***	5
Perrin et al., 1999[72]	*		***	4
Rahal et al., 2015[58]			**	2
Tsang & Hui-Chan, 2004 [59]	***		***	6
Tsang & Hui-Chan, 2005 [60]	***	*	***	7
Tsang et al., 2004 [66]	***		***	6
Tsang & Hui-Chan, 2006 [61]	***	*	***	7
Tsang & Hui-Chan, 2010 [62]	***	*	***	7
Wayne et al., 2014 [49]	***	*	***	7
Wong et al., 2001 [67]	*	*	***	4
Wong et al., 2011 [68]		*	***	4
Zhang et al., 2011 [63]		*	***	4

#### Effects of more PA versus less PA

##### Primary outcomes

(indirect measures of balance). Initial analyses included 16 variables (20 studies; *n* = 1053 participants) (Table 3). Sensitivity analysis removed five variables (which are excluded from Table 3) due to their high risk of bias (maximal walking speed, functional reach in back, left and right directions, and range of motion), resulting in only 11 variables (13 studies; 733 participants).

**Table 3:** Primary outcomes - more active versus less active groups (Indirect measures of balance)

Comparison or subgroup	No. of studies	N	Effect size (95% CI)	Heterogeneity
Neuromuscular measure of gait				
*1 Preferred walking speed (m/s).	4	284	0.24 (−0.69, 1.17)	91%
Preferred walking speed (m/s).	2	194	0.66 (0.26, 1.06)	20%
Neuromuscular measures of strength				
*2 Handgrip (Kg). ++	2	210	1.73 (−1.20, 4.66)	23%
*3 Isometric knee extension.	4	320	0.63 (0.40, 0.87)	35%
3.1 Isometric knee extension.	3	292	0.64 (0.35, 0.94)	25%
*4 Ultrasound.	2	158	0.57 (0.25, 0.89)	0%
Neuromuscular measures of functionality				
*5 Timed Up & Go. (s) Low value indicates better balance.	4	286	−0.76 (−1.01, −0.51)	0%
5.1 Timed Up & Go. (s) Low value indicates better balance.	2	161	−0.70 (−1.03, − 0.37)	0%
*6 Single Leg Stance. (s)	4	181	−0.25 (−1.86, 1.37)	95%
6.1 Single Leg Stance. (s)	2	110	1.17 (0.74, 1.60)	0%
*7 Activities of Balance Confidence.	4	220	1.33 (0.73, 1.94)	74%
7.1 Activities of Balance Confidence.	3	155	1.47 (0.70, 2.25)	70%
Neuromuscular measures of flexibility				
*8 Functional reach (forward) (m).	4	304	1.18 (0.61, 1.75)	74%
8.1 Functional reach (forward) (m).	2	193	0.80 (0.48, 1.11)	0%
Sensory measures				
*9 Knee joint repositioning (degrees).	2	58	−1.37 (−2.29, −0.45)	59%
Cognitive measures				
*10 Mini Mental State Exam. ++	4	229	0.37 (−0.35, 1.09)	60%
*11 Reaction time (s). Low value indicates better balance.	3	198	−0.75 (−1.45, − 0.04)	83%
11.1 Reaction time (s). Low value indicates better balance.	2	132	−0.41(− 0.84, 0.01)	33%

Note: Data is shown for 11 variables. For some variables there are two sets of data, the first set of data identified with * includes all available data, whereas the second set of data excludes studies at high risk of bias

Analyses with <2 studies providing data are not shown (maximal walking speed, functional reach (back, left, right), and range of motion are excluded)

Higher value indicates better balance unless otherwise stated

++ Mean difference (95% CI) was calculated (MMSE and Handgrip test) and standardised mean (95% CI) calculated for all other measures.

Sensitivity analyses showed significant differences between more and less active groups for two variables (preferred walking speed and SLS), which were not identified in initial analyses, but otherwise did not alter findings (Table 3).

##### Neuromuscular measures

Table 3 shows that more active groups achieved faster gait speed (SMD 0.66 m/s); better results for two measures of strength using ultra sound tests (SMD 0.57) and isometric knee extension tests (SMD 0.64); better results for three measures of functionality with longer time on SLS test (SMD 1.17s), higher scores on ABC (SMD 1.47), and faster time taken to complete the TUG test (SMD − 0.70s); and better results for one measure of flexibility with greater distances achieved for the functional reach test (forward) (SMD 0.80m).

##### Sensory measures

Less active groups achieved statistically significant better results for one sensory measure of balance with better results on knee joint repositioning tests (SMD − 1.37).

There was no statistically significant difference between more active and less active groups for neuromuscular measures such as handgrip strength or cognitive measures such as MMSE scores or reaction time.

##### Secondary outcomes

(direct measures of balance). Twelve variables were included in analyses (14 studies; *n* = 801 participants) (Table 4: analyses highlighted*). However, for sensitivity analyses three studies were removed, due to high risk of bias (*n* = 162 participants) leaving ten variables (11 studies; *n* = 639 participants) for analysis: significance levels decreased for static body stability eyes open and eyes closed (speed).

**Table 4:** Secondary outcomes - more active versus less active groups (Direct measures of balance)

Comparison or subgroup	No. of studies	N	Effect size	Heterogeneity
*1 Somatosensory Organisation Test (Somatosensory. ratio).++	3	139	0.90 (−0.58, 2.38)	81%
1.1 Somatosensory Organisation Test (Somatosensory. ratio). ++.	2	63	0.16 (003, 0.29)	0%
*2 Somatosensory Organisation Test (Visual ratio). ++	3	139	−2.71 (−3.99, −1.44)	100%
2.1 Somatosensory Organisation Test (Visual ratio). ++	2	63	0.13 (0.03, 0.22)	40%
*3 Somatosensory Organisation Test (Vestibular ratio). ++	3	139	−0.02 (−0.04, 0.00)	0%
3.1 Somatosensory Organisation Test (Vestibular ratio). ++	2	63	−0.02 (− 0.04, 0.00)	0%
*4 Static total body stability eyes open (m). Low value indicates better balance.	3	302	−0.37 (− 0.74, 0.01)	57%
*5 Static total body stability eyes open (cm^2^). Low value indicates better balance.	4	231	−0.89 (−2.11, 0.33)	93%
5.1 Static total body stability eyes open (cm^2^). Low value indicates better balance.	2	145	0.34 (−0.25, 0.94)	66%
*6 Static total body stability eyes open (velocity) (cm/s). Low value indicates better balance.	3	161	−1.55 (−3.35, 0.25)	95%
6.1 Static total body stability eyes open (velocity) (cm/s). Low value indicates better balance.	2	135	0.07 (−0.29, 0.43)	2%
*7 Static total body stability eyes closed (velocity) (cm/s). Low value indicates better balance.	3	161	−1.67 (−3.50, 0.16)	95%
7.1 Static total body stability eyes closed (velocity) (cm/s). Low value indicates better balance.	2	135	−3.05 (−9.53, 3.43)	2%
*8 Static ML stability body angle (degrees). Low value indicates better balance.	2	96	−0.12 (−0.52, 0.28)	0%
*9 Static AP stability body angle (degrees). Low value indicates better balance.	2	96	−0.11 (− 0.75, 0.53)	60%
*10 Dynamic AP stability (forward) (angle °). Low value indicates better balance.	2	72	0.01 (−2.19, 2.22)	94%
*11 Dynamic Loss of Stability (max excursion) (%). Low value indicates better balance.	2	68	1.09 (0.57,1.60)	0%
*12 Dynamic Loss of stability (directional control) (%). Low value indicates better balance.	2	68	1.02 (0.47, 1.58)	11%

Note: Data is shown for 12 variables. For some variables there are two sets of data, the first set of data identified with * includes all available data, whereas the second set of data excludes studies at high risk of bias

Higher value indicates better balance unless otherwise stated

++ Mean difference (95% CI) was calculated (SOT visual, vestibular and somatosensory ratios), and standardised mean (95% CI) calculated for all other measures

More active groups achieved statistically significant better results in three secondary outcome measures, with better tilt board results on directional control (SMD 1.02), and maximum excursion (SMD 1.09) as well as SOT visual ratios (SMD 0.13).

There was no statistically significant difference between more and less active groups for other measures of static or dynamic balance.

### Intervention studies

#### Design, sample size, and location

Due to the inclusion criteria only four randomised controlled trials (RCTs) were included [49, 73–75]. Sample size ranged from 20 [74] to 60 [49] with an average of 38 participants, and only one study [49] justified sample size.

Of the four studies, one was US based [49] and the country for the remainder was not specified.

#### Participants

Participants across all studies were defined as healthy and resided in the community (62% women; mean age = 68.78 years), but there was a lack of more detailed demographic information. Average age of participants was 61–70 years in three studies [49, 73, 74], and 71 years or over in one study [75].

#### Physical activity

All studies included a less active group and a more active group, and all PA interventions were land based where two included ‘3D PA’ (*n* = 109 participants) (Tai Chi) [49, 75], and two included ‘General PA’ (*n* = 41 participants) (walking) [73, 74]. Only one study used a validated PA assessment tool used (e.g. PASS) [49].

Intervention duration ranged from a minimum of three months [73, 74] to a maximum of six months [49, 75]. All four provided results at baseline and post-trial commencement, at three months [73], at four months [74], at both two and six months [75], and at both three and six months [49].

#### Balance

All studies included a neuromuscular balance measure, but only one included a measure of the cognitive system (MMSE) [49], and none included any sensory system measures.

*Secondary outcome measures.*

One study used the SOT [75], and three used force plate platforms [49, 73, 74].

#### Quality

**Fig. 2:**
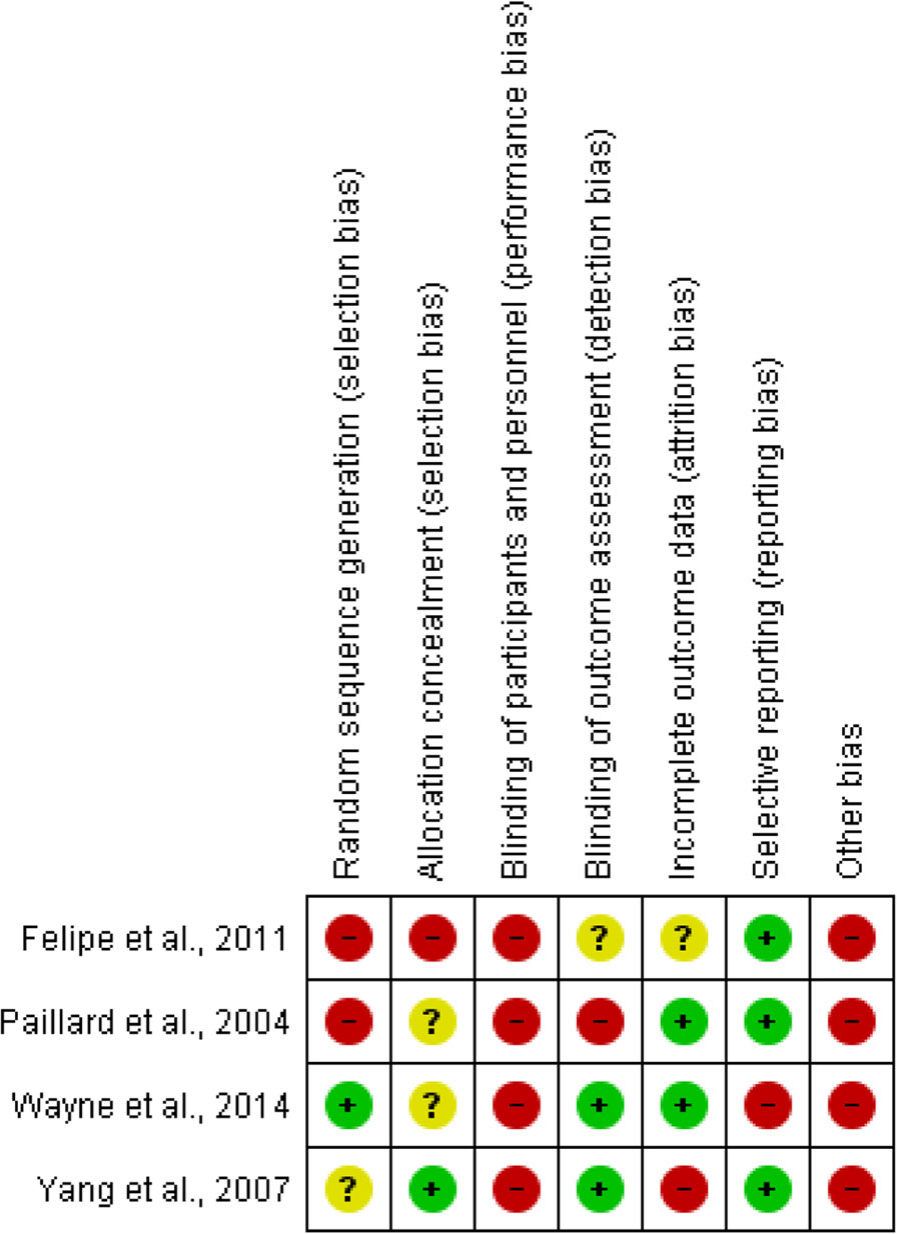
A summary table of review authors’ judgements for each risk of bias item for each study

Figure 2 presents a summary table of the risk of bias of included intervention studies, and shows a high risk of bias for all studies.

#### Effects of more PA versus less PA

Due to the limited number of studies and lack of common outcomes, a best evidence synthesis was explored [46].

##### Key findings relating to direct measures of balance

Two studies reported direct measures [49, 73], but only one study provided these measures post-intervention measuring neuromuscular system health using gait speed only [73],and found that walking improved gait speed in more active groups. However, the study was at high risk of bias [29] and of low methodological quality (level 3) [46] and so provides limited evidence.

##### Key findings relating to secondary measures of balance

All four studies reported secondary measures of balance (e.g. SOT vestibular, BoS, and static and dynamic balance), and found that intervention groups had better balance scores. However, all studies were at high risk of bias [29] and of low methodological quality [46], and so evidence is again limited.

##### Key findings overall

There is limited evidence that free-living PA improves measures of balance in older healthy community-dwelling adults.

#### Subgroup analyses

The heterogeneity in the nature of the outcome data relating to age, type of PA and duration of effect meant that it was not possible to explore the effects of PA in relation to these variables.

## Discussion

This review explored the role of free-living PA in relation to balance outcomes across multiple body systems, and summarises two types of evidence. The majority of evidence was from cross sectional studies (26 studies) of moderate methodological quality, and a much smaller number was from RCTs (four studies) of low methodological quality.

The evidence from cross sectional studies found that free-living PA [25–27] is beneficial for balance in older healthy community-dwelling adults (50 years and over), where more active groups experienced better performance on indirect measures of gait speed, strength, functionality and flexibility, and on direct measures of directional control, maximum excursion and SOT visual ratios. These findings extend the results from a previous longitudinal research exploring PA and physical performance by Cooper et al., that found that leisure-time PA carried out over the longer-term (17 years) can improve neuromuscular measures of strength in middle-aged adults (36-53 yrs) [76]. Additionally, evidence from the limited number of RCTs suggests that free-living PA improves measures of balance in the short-term (three-six months) in older healthy community-dwelling adults which extends the findings from previous research, that short-term (three-six months) exercise, a sub-category of PA, improves balance performance in older unhealthy adults [8, 13].

It is evident from this study that few RCTs have explored free-living PA and balance and that most evidence has been derived from observational studies, thus potentially providing insufficient clinical trial data on which to base clear conclusions. However, research suggests that the effects of free-living PA require a longer duration of study than that afforded by RCTs [77]. This review included observational studies that explored free-living PA of between one and 21 years’ duration. In contrast, Howe et al.’s [13] systematic review of RCTs found no evidence that free-living PA such as walking or cycling, of up to 6 months’ duration, improved measures of balance in older unhealthy adults. Thus, the benefits realised from free-living PA may be cumulative over time, and future research should consider the appropriateness of the study design involved in exploring associations between free-living PA and balance.

A strength of this review is that it considers balance as a multidimensional construct [1, 3] rather than a single system, and as a result, includes measures across neuromuscular, cognitive and sensory body systems, thus measures balance holistically. However, it is evident that whilst this review sought to include measures from multiple body systems, the majority of studies focused on neuromuscular measures (19 of 30 studies) and a smaller number included cognitive (ten) measures, and even less included sensory measures (three). Additionally, this study found no effect for cognitive measures relating to PA level, and this may be due to the inclusion of healthy older adults in the present study. As a result, future studies should seek to include measures across all the body systems required for balance, and include unhealthy adults.

Studies in the review reported validated measures for both balance and PA. Whilst most measures of PA were subjective, except for those in one study [47], the balance measures included were mainly objective, thus reducing any measurement bias due to self-reporting and or recall bias in the results [78].

There are some limitations to be taken into account when considering these findings. For example, sample size for both cross-sectional studies and RCTs were small ranging from 20 to 170 participants, and only justified by a power calculation in one study [49] which may give rise to Type II errors. Additionally, the observational studies included were cross sectional studies and therefore no causal relationship between free-living PA and balance can be determined. Also, participants were either volunteers or recruited using convenience sampling, therefore the generalisability of the findings is limited. In addition, whilst this review included multiple balance measures across different body systems, the number of different outcome measures (*n* = 40) restricted the ability to compare and pool results, and therefore future research in this emerging area should consider establishing a consensus of relevant balance measures across all body systems to aid analysis and fully understand the effects of free-living PA on balance.

In summary, this review suggests that free-living PA improves balance performance in older healthy adults both in the short-term and long-term using validated and objective measures across multiple body systems. Further research that incorporates higher quality studies is warranted, with the inclusion of longitudinal studies that provide large samples of participants using robust selection processes, and appropriate data over multiple time points. For example, studies such as NICOLA (Northern Ireland Cohort of Longitudinal Ageing) [79], TILDA (The Irish Longitudinal Study of Ageing) [32], and ELSA (English Longitudinal Study of Ageing) [31] include large samples of community-dwelling participants (50 years and over) (8500, 8504 and 11, 391 respectively); provide data across multiple timepoints (between three and 11 years); adhere to the Gateway to Global Ageing Initiative [80] which improves the harmonisation of balance outcomes, therefore reducing the variability of outcomes and improving comparability of results; and include balance measures across multiple body systems that are objective and validated.

## Conclusion

In conclusion, there is limited evidence from a small number of RCTs, and moderate quality of evidence from observational studies that suggests that free-living PA improves measures of balance in older community-dwelling healthy adults, particularly in respect of fall prevention. Future research should consider longitudinal studies of good methodological quality to improve the overall robustness of the findings.

## Additional files

Additional file 1: Medline search example. (DOCX 12 kb)

Additional file 2: Table showing characteristics of excluded studies. (DOCX 27 kb)

## Abbreviations

ABC: Activities of Balance Confidence
AMED: Allied and Complementary Medicine Database
AP: Anterior Posterior
CDSR: Cochrane Database of Systematic Reviews
CENTRAL: Central Register of Controlled Trials
CI: Confidence Interval
CINAHL: Cumulative Index to Nursing and Allied Health Literature
COP: Centre of Pressure
ELSA: English Longitudinal Study of Ageing
MEDLINE: Medical Literature Analysis and
ML: Medio Lateral
MLTPAQ: Minnesota Leisure Physical Activity Questionnaire
MMSE: Mini Mental State Exam
NOS: Newcastle Ottawa Scale
PA: Physical Activity (Free-living PA is activity for leisure, travel, occupational, or exercise)
PASS: Physical Activity Status Score
PRISMA: Preferred Reporting Items for Systematic Reviews and Meta-Analyses
RAPA: Rapid Assessment of Physical Activity
RCT: Randomised Controlled Trials
SLS: Single Leg Stance
SMD: Standardised Mean Difference
SOT: Sensory Organisation Test
TILDA: The Irish Longitudinal Study on Ageing
TUG: Timed up and Go test
