# Supplementary material for: The association between balance and free-living physical activity in an older community-dwelling adult population: a systematic review and meta-analysis
Source: BMC Public Health. 2018 Apr 2;18:431. doi: 10.1186/s12889-018-5265-4 (PMC5879995; doi:10.1186/s12889-018-5265-4)
Supplement: Supplementary file 1 — Medline search example. (DOCX 12 kb) [file 12889_2018_5265_MOESM1_ESM.docx]

**OVID MEDLINE (R) 1946 to May Week 1 2016 - 643**

1 musculoskeletal physiological phenomena/ or postural balance/

2 Accidental Falls/

3 1 or 2

4 human activities/ or "activities of daily living"/ or exercise/ or leisure activities/ or travel/ or work/

5 movement/ or gait/ or running/ or swimming/ or walking/ or physical endurance/ or physical fitness/

6 4 or 5

7 3 and 6

8 (healthy not (amput* or arthriti* or osteoporos* or "musc* dis*" or "nerv* system dis*" or "neur* dis*" or Alzheimer* or Parkinson* or dementia* or "multiple sclerosis*" or "somatosensory* dis*" or "hear* dis*" or "vis* dis*" or "history of fall*" or "history of fracture*")).mp. [mp=title, abstract, original title, name of substance word, subject heading word, keyword heading word, protocol supplementary concept word, rare disease supplementary concept word, unique identifier]

9 7 and 8

10 limit 9 to (english language and ("middle age (45 to 64 years)" or "middle aged (45 plus years)" or "all aged (65 and over)" or "aged (80 and over)"))
