# Supplementary material for: The association between balance and free-living physical activity in an older community-dwelling adult population: a systematic review and meta-analysis
Source: BMC Public Health. 2018 Apr 2;18:431. doi: 10.1186/s12889-018-5265-4 (PMC5879995; doi:10.1186/s12889-018-5265-4)
Supplement: Supplementary file 2 — Table showing characteristics of excluded studies. (DOCX 27 kb) [file 12889_2018_5265_MOESM2_ESM.docx]

Characteristics of excluded studies table

| Full reference | Author | No balance measure | No PA measure | Age | Structured exercise/lab based | Unhealthy population | No comparison group | Study type | other |
| --- | --- | --- | --- | --- | --- | --- | --- | --- | --- |
| Akosile, C.O., Anukam, G.O., Johnson, O.E., Fabunmi, A.A., Okoye, E.C., Iheukwumere, N. and Akinwola, M.O. (2014) Fear of falling and quality of life of apparently-healthy elderly individuals from a Nigerian population. *Journal of Cross-Cultural Gerontology,* 29(2), 201-209. | Akosile et al., 2014 | X |  |  |  |  |  |  |  |
| Albert, S.M., King, J., Boudreau, R., Prasad, T., Lin, C.J. and Newman, A.B. (2014) Primary prevention of falls: effectiveness of a statewide program. *American Journal of Public Health,* 104(5), e77-84. | Albert et al., 2014 |  | X |  |  | X |  |  |  |
| Alexander, N.B. and Hausdorff, J.M. (Dec 2008) Linking thinking, walking, and falling. *The Journals of Gerontology: Series A: Biological Sciences and Medical Sciences,* 63A(12), 1325-1328. | Alexander et al., 2008 |  |  |  |  |  |  | X  Discussion paper-no results |  |
| Alpert, P.T., Miller, S.K., Wallmann, H., Havey, R., Cross, C., Chevalia, T., Gillis, C.B. and Kodandapari, K. (2009) The effect of modified jazz dance on balance, cognition, and mood in older adults. *Journal of the American Academy of Nurse Practitioners,* 21(2), 108-115 8p. | Alpert et al., 2009 |  |  |  |  |  | X |  |  |
| Aranda-García, S., Busquets, A., Planas, A., Prat-Subirana, J. and Angulo-Barroso, R. (2015) Strength, Static Balance, Physical Activity, and Age Predict Maximal Gait Speed in Healthy Older Adults From a Rural Community: A Cross-Sectional Study. *Journal of Aging & Physical Activity,* 23(4), 580-587 8p. | Aranda-Garcia et al., 2015 |  |  |  |  |  | X |  |  |
| Reference |  | No balance measure | No PA measure | Age | Structured exercise/lab based | Unhealthy population | No comparison group | Study type | other |
| Bauman, A.E. and Smith, B.J. (2000) Healthy ageing: what role can physical activity play? *Medical Journal of Australia,* 173(2), 88-90. | Bauman et al., (2000) |  |  |  |  |  |  | X  Discussion paper |  |
| Brouwer, B., Musselman, K. and Culham, E. (2004) Physical function and health status among seniors with and without a fear of falling. *Gerontology,* 50(3), 135-141. | Brouwer et al., (2004) |  | X |  |  | X  Fall history |  |  |  |
| Buatois S. Gauchard GC. Aubry C. Benetos A. Perrin,P. (2007) Current Physical Activity Improves Balance Control during Sensory Conflicting Conditions in Older Adults. *International Journal of Sports Medicine,* 28(1), 53-58. | Buatois et al., (2007) |  |  |  |  | X  Fall history |  |  |  |
| Busing, J.K. (2005) Determining the effects of Tai Chi on dynamic balance and fear of falling in an elderly population. *Activities, Adaptation & Aging,* 30(2), 49-64 16p. | Busing, (2005) |  |  |  |  | X  Fall history |  |  |  |
| Cancela Carral, J.M., Romo Pérez, V. and Camiña Fernández, F. (2002) Healthy physical activity for elders. Condition parameters. *Gerokomos,* 13(4), 191-203 13p. | Cancela et al., (2002) |  |  |  |  |  |  |  | X  Non-english |
| Dattilo, J., Martire, L. and Proctor, D. (2012) B-Active: An Interdisciplinary Approach to Healthy Aging. *Therapeutic Recreation Journal,* 46(3), 191-201 11p. | Dattilo et al., (2012) |  |  |  |  | X  History of falls |  |  |  |
| de Rekeneire, N., Visser, M., Peila, R., Nevitt, M.C., Cauley, J.A., Tylavsky, F.A., Simonsick, E.M. and Harris, T.B. (2003) Is a fall just a fall: correlates of falling in healthy older persons. The Health, Aging and Body Composition Study. *Journal of the American Geriatrics Society,* 51(6), 841-846. | De Rekeneire et al., (2003) |  | X  physical capability. |  |  |  |  |  |  |
| Reference |  | No balance measure | No PA measure | Age | Structured exercise/lab based | Unhealthy population | No comparison group | Study type | other |
| Demura S., Yamada T. and Kasuga, K. (2012) Severity of injuries associated with falls in the community dwelling elderly are not affected by fall characteristics and physical function level. *Archives of Gerontology and Geriatrics,* 55(1), 186-189. | Demura et al., (2012) |  | X |  |  |  |  |  |  |
| Domaradzki A. Ignasiak Z. Stodolka,W. (2014) The predictors of falls in adult and senior women from cities of lower silesia, Poland. *Human Movement Science,* 15(2), 86-92. | Domaradzki et al., (2014) |  | X |  |  |  | X |  |  |
| Earles, D.R., Judge, J.O. and Gunnarsson, O.T. (2001) Velocity training induces power-specific adaptations in highly functioning older adults. *Archives of Physical Medicine & Rehabilitation,* 82(7), 872-878. | Earles et al., (2001) |  |  |  |  | X  Arthritis still include |  |  |  |
| Egerton, T., Brauer, S.G. and Cresswell, A.G. (2009) Fatigue after physical activity in healthy and balance-impaired elderly. *Journal of Aging and Physical Activity,* 17(1), 89-105. | Egerton et al., (2009) |  | X |  |  | X  Balance impaired |  |  |  |
| Ekman, G. and Gard, G. (2001) [Functional test related to the demands of cleaners' work]. *Nordisk Fysioterapi,* 5(2), 68-74 7p. | Ekman et al., (2001) |  |  |  |  |  |  |  | X  Not available |
| El Haber, N., Erbas, B., Hill, K.D. and Wark, J.D. (2008) Relationship between age and measures of balance, strength and gait: linear and non-linear analyses. *Clinical Science,* 114(12), 719-727. | El Haber et al., (2008) |  |  |  |  | X  History of falls | X  Split by age |  |  |
| Faude, O., Donath, L., Roth, R., Fricker, L. and Zahner, L. (2012) Reliability of gait parameters during treadmill walking in community-dwelling healthy seniors. *Gait & Posture,* 36(3), 444-448. | Faude et al., (2012) |  |  |  |  | X  History of falls |  |  |  |
| Reference |  | No balance measure | No PA measure | Age | Structured exercise/lab based | Unhealthy population | No comparison group | Study type | other |
| Frandin, K., Sonn, U., Svantesson, U. and Grimby, G. (1995) Functional balance tests in 76-year-olds in relation to performance, activities of daily living and platform tests. *Scandinavian Journal of Rehabilitation Medicine,* 27(4), 231-241. | Frandin et al., (1995) |  | X |  |  |  |  |  |  |
| Freitas, M.d.B.Z., Mauerberg-Decastro, E. and Moraes, R. (2013) Intermittent use of an 'anchor system' improves postural control in healthy older adults. *Gait & Posture,* 38(3), 433-437 5p. | Freitas et al., (2013) |  | X |  |  |  |  |  |  |
| Gill, T.M., Pahor, M., Guralnik, J.M., McDermott, M.M., King, A.C., Buford, T.W., Strotmeyer, E.S., Nelson, M.E., Sink, K.M., Demons, J.L., Kashaf, S.S., Walkup, M.P., Miller, M.E. and LIFE Study Investigators. (2016) Effect of structured physical activity on prevention of serious fall injuries in adults aged 70-89: Randomized clinical trial (LIFE Study). *BMJ: British Medical Journal,* 352 Feb, Art 245-8. | Gill et al., (2016) |  |  |  |  | X  Functional disabilities |  |  |  |
| Goutier, K.M.T., Jansen, S.L., Horlings, C.G.C., Kung, U.M. and Allum, J.H.J. (2010) The influence of walking speed and gender on trunk sway for the healthy young and older adults. *Age & Ageing,* 39(5), 647-650 | Goutier et al., (2010) |  | X |  |  |  |  |  |  |
| Graafmans, W.C., Lips, P., Wijlhuizen, G.J., Pluijm, S.M. and Bouter, L.M. (2003) Daily physical activity and the use of a walking aid in relation to falls in elderly people in a residential care setting. *Zeitschrift Fur Gerontologie Und Geriatrie,* 36(1), 23-28. | Graafmans et al., (2003) |  | X |  |  | X  Fall history |  |  |  |
| Groessl, E.J., Kaplan, R.M., Rejeski, W.J., Katula, J.A., King, A.C., Frierson, G., Glynn, N.W., Hsu, F.C., Walkup, M. and Pahor, M. (2007) Health-related quality of life in older adults at risk for disability. *American Journal of Preventive Medicine,* 33(3), 214-218. | Gressl et al., (2007) | X |  |  |  |  | X |  |  |
| Reference |  | No balance measure | No PA measure | Age | Structured exercise/lab based | Unhealthy population | No comparison group | Study type | other |
| Guan H & Koceja DM (2011). Effects of long-term tai chi practice on balance and H-refex characteristics. Americain Journal of Chinese Medicine, 39(2) 251-260 | Guan et al., (2011) |  |  | X |  |  |  |  |  |
| Gudlaugsson, J., Aspelund, T., Gudnason, V., Olafsdottir, A.S., Jonsson, P.V., Arngrimsson, S.A. and Johannsson, E. (2013) The effects of 6 months' multimodal training on functional performance, strength, endurance, and body mass index of older individuals. Are the benefits of training similar among women and men? *Læknabla?i?,* 99(7-8), 331-337. | Gudlaugsson et al., (2013) |  |  |  | X  Walking performed indoors & include endurance training |  |  |  |  |
| Gustafson, A.S., Noaksson, L., Kronhed, A.C., Moller, M. and Moller, C. (2000) Changes in balance performance in physically active elderly people aged 73-80. *Scandinavian Journal of Rehabilitation Medicine,* 32(4), 168-172. | Gustafson et al., (2000) |  |  |  |  | X  Hospitalized population |  |  |  |
| Halvarsson, A., Olsson, E., Faren, E., Pettersson, A. and Stahle, A. (2011) Effects of new, individually adjusted, progressive balance group training for elderly people with fear of falling and tend to fall: A randomized controlled trial. *Clinical Rehabilitation,* 25(11), 1021-1031. | Halvarsson et al., (2011) |  |  |  |  | X  Fall history |  |  |  |
| Halvarsson, A., Franzén, E., Farén, E., Olsson, E., Oddsson, L. and Ståhle, A. (2013) Long-term effects of new progressive group balance training for elderly people with increased risk of falling - a randomized controlled trial. *Clinical Rehabilitation,* 27(5), 450-458. | Halvarsson et al., (2013) |  |  |  |  | X  Fall history |  |  |  |
| Reference |  | No balance measure | No PA measure | Age | Structured exercise/lab based | Unhealthy population | No comparison group | Study type | other |
| Halvarsson, A., Olsson, E., Elin, F., Pettersson, A. and Stahle, A. (2011) A new individually adjusted, progressive balance group training program for elderly with fear of falling and a tendency to fall. *Physiotherapy (United Kingdom),* 97, eS448-eS449. | Halvarsson et al., (2011) |  |  |  |  | X  Fall history |  |  |  |
| Heesch, K.C., Byles, J.E. and Brown, W.J. (2008) Prospective association between physical activity and falls in community-dwelling older women. *Journal of Epidemiology & Community Health,* 62(5), 421-426. | Heesch et al., (2008) |  |  |  |  | X  Fall history |  |  |  |
| Karinkanta S., Heinonen A., Sievanen H., UusiRasi K. and Kannus, P. (2005) Factors predicting dynamic balance and quality of life in home-dwelling elderly women. *Gerontology,* 51(2), 116-121. | Karinkanta et al., (2005) |  |  |  |  | X | X |  |  |
| Kelsey J.L., Berry S.D., ProcterGray E., Quach L., Nguyen U.S.D.T., Li W., Kiel D.P., Lipsitz L.A. and Hannan, M.T. (2010) Indoor and outdoor falls in older adults are different: The maintenance of balance, independent living, intellect, and zest in the elderly of boston study. *Journal of the American Geriatrics Society,* 58(11), 2135-2141. | Kelsey et al., (2010) |  |  |  |  | X  Fall history |  |  |  |
| Kermode-Scott, B. (2002) Healthy aging. *Canadian Family Physician,* 48(JAN.) (pp 213-214), ate of Pubaton: 2002. | Kermode-Scott, (2002) |  |  |  |  |  |  | X Discussion paper |  |
| Kim WJ. Chang M. An,D.H. (2014) Effects of a community-based fall prevention exercise program on activity participation. *Journal of Physical Therapy Science,* 26(5), 651-653. | Kim et al., (2014) |  |  |  |  |  | X |  |  |
| Kolt, G., Schofield, G.M., Kerse, N., Garrett, N. and Ashton, T. (2011) Healthy steps trial: Effectiveness of a pedometer-based green prescription for low-active older adults in primary care. *Physiotherapy (United Kingdom),* 97, eS630. | Kolt et al., (2011) |  |  |  |  |  |  | X  Protocol |  |
| Reference |  | No balance measure | No PA measure | Age | Structured exercise/lab based | Unhealthy population | No comparison group | Study type | other |
| Kramer, B.J., Creekmur, B., Mitchell, M.N., Rose, D.J., Pynoos, J. and Rubenstein, L.Z. (2014) Community fall prevention programs: comparing three InSTEP models by levels of intensity. *Journal of Aging & Physical Activity,* 22(3), 372-379. | Kramer et al., (2014) |  |  |  | X  Flexibility training |  |  |  |  |
| Melo, C., Kuisma, R. and Trew, M. (2011) The impact of a specific, moderate and safe home-based exercise programme on fall risk factors in older portuguese people. *Physiotherapy (United Kingdom),* 97, S799. | Melo et al., (2011) |  |  |  | X  Walking is part of the exercise programme but includes strengthening training exercise |  |  |  |  |
| Melzer, I., Benjuya, N. and Kaplanski, J. (2003) Effects of regular walking on postural stability in the elderly. *Gerontology,* 49(4), 240-245. | Melzer et al., (2003) |  |  |  |  |  |  | X  Retrospective case control study |  |
| Melzer, I. and Oddsson, L. (2013) Improving balance control and self-reported lower extremity function in community-dwelling older adults: a randomized control trial. *Clinical Rehabilitation,* 27(3), 195-206. | Melzer et al., (2013) |  |  |  |  |  |  | X  Retrospective case control study |  |
| Reference |  | No balance measure | No PA measure | Age | Structured exercise/lab based | Unhealthy population | No comparison group | Study type | other |
| Mendoza-Ruvalcaba, N.M. and Arias-Merino, E.D. (2015) "I am active": effects of a program to promote active aging. *Clinical Interventions in Aging,* 10, 829-837. | Mendoza-Ruvalcaba & Arias-Merino(2015) |  |  |  | X  Strength and balance exercise |  |  |  |  |
| Meuleman, J.R., Hoffman, N.B., Conlin, M.M., Lowenthal, D.T., Delafuente, J.C. and Graves, J.E. (1992) Health status of the aged: medical profile of a group of functional elderly. *Southern Medical Journal,* 85(5), 464-468. | Meuleman et al., (1992) |  |  |  |  | X |  | X  Retrospective analysi |  |
| MonteroOdasso M., Schapira M., Soriano E.R., Varela M., Kaplan R., Camera L.A. and Mayorga, L.M. (2005) Gait velocity as a single predictor of adverse events in healthy seniors aged 75 years and older. *Journals of Gerontology - Series A Biological Sciences and Medical Sciences,* 60(10), 1304-1309. | Montero-Odasso et al., 2005 |  | X |  |  |  |  |  |  |
| Musselman, K. and Brouwer, B. (Jul 2005) Gender-Related Differences in Physical Performance Among Seniors. *Journal of Aging and Physical Activity,* 13(3), 239-253. | Musselman et al (2005) |  | X |  |  |  | X |  |  |
| Paterson, K., Hill, K. and Lythgo, N. (2011) Stride dynamics, gait variability and prospective falls risk in active community dwelling older women. *Gait & Posture,* 33(2), 251-255. | Paterson et al (2011) |  | X |  |  |  |  |  |  |
| Pober, D.M., Freedson, P.S., Kline, G.M., McInnis, K.J. and Rippe, J.M. (2002) Relationship of age to selected fitness and health related measures in healthy adults ages 40 to 79 years. *Clinical Exercise Physiology,* 4(2), 108-119 12p. | Pober et al (2002) |  |  |  |  |  | X |  |  |
| Reference |  | No balance measure | No PA measure | Age | Structured exercise/lab based | Unhealthy population | No comparison group | Study type | other |
| Rosano, C., Simonsick, E.M., Harris, T.B., Kritchevsky, S.B., Brach, J., Visser, M., Yaffe, K. and Newman, A.B. (2005) Association between physical and cognitive function in healthy elderly: the health, aging and body composition study. *Neuroepidemiology,* 24(1-2), 8-14. | Rosano et al (2005) |  |  |  |  |  | X |  |  |
| Tsang W.W.N., Gao K.L. and HuiChan, C.W.Y. (2011.) Effects of golfing on postural control and confidence in older adults. *Physiotherapy (United Kingdom),* 97, eS1255-eS1256. | Tsang et al (2011) |  |  |  |  |  |  |  | X  No UK locations |
| Valentine, R.J., Misic, M.M., Rosengren, K.S., Woods, J.A. and Evans, E.M. (2009) Sex impacts the relation between body composition and physical function in older adults. *Menopause,* 16(3), 518-523. | Valentine et al (2009) |  |  |  |  |  | X |  |  |
| Van Dijk GP, Lenssen AF, Kingma H & Lodder J. (2013) Taekwondo traininf improves balance in volunteers over 40. Frontiers in Aging Neuroscience. Volume 5 Article 10 1-6 | Van Dijk et al., 2013 |  |  |  |  |  | X |  |  |
| Yamada, T. and Demura, S. (2009) Relationships between ground reaction force parameters during a sit-to-stand movement and physical activity and falling risk of the elderly and a comparison of the movement characteristics between the young and the elderly. *Archives of Gerontology and Geriatrics,* 48(1), 73-77. | Yamada et al (2009) |  |  |  |  |  | X |  |  |
